# Which long noncoding RNAs and circular RNAs contribute to inflammatory bowel disease?

**DOI:** 10.1038/s41419-020-2657-z

**Published:** 2020-06-15

**Authors:** Lihui Lin, Gaoshi Zhou, Peng Chen, Ying Wang, Jing Han, Minhu Chen, Yao He, Shenghong Zhang

**Affiliations:** Division of Gastroenterology, The First Affiliated Hospital, Sun Yat-sen University, Guangzhou, P. R. China

**Keywords:** Inflammatory bowel disease, Chronic inflammation

## Abstract

Inflammatory bowel disease (IBD), a chronic relapsing gastrointestinal inflammatory disease, mainly comprises ulcerative colitis (UC) and Crohn’s disease (CD). Although the mechanisms and pathways of IBD have been widely examined in recent decades, its exact pathogenesis remains unclear. Studies have focused on the discovery of new therapeutic targets and application of precision medicine. Recently, a strong connection between IBD and noncoding RNAs (ncRNAs) has been reported. ncRNAs include microRNAs (miRNAs), long noncoding RNAs (lncRNAs), and circular RNAs (circRNAs). The contributions of lncRNAs and circRNAs in IBD are less well-studied compared with those of miRNAs. However, lncRNAs and circRNAs are likely to drive personalized therapy for IBD. They will enable accurate diagnosis, prognosis, and prediction of therapeutic responses and promote IBD therapy. Herein, we briefly describe the molecular functions of lncRNAs and circRNAs and provide an overview of the current knowledge of the altered expression profiles of lncRNAs and circRNAs in patients with IBD. Further, we discuss how these RNAs are involved in the nosogenesis of IBD and are emerging as biomarkers.

**Facts**

- long noncoding RNAs (lncRNAs) and circular RNAs (circRNAs) are involved in the pathogenesis of inflammatory bowel disease (IBD).
- Moreover, certain lncRNAs and circRNAs are potential biomarkers of IBD.
- The contributions of lncRNAs and circRNAs in IBD will become hot spots in future studies.

**Open questions**

- lncRNAs and circRNAs show altered expression profile in patients with IBD compared with those in healthy controls.
- Which and how are lncRNAs and circRNAs involved in the internal mechanism of IBD?
- Will lncRNAs and circRNAs serve as clinical biomarkers of IBD?

## Introduction

Inflammatory bowel disease (IBD) is a chronic inflammatory disease within the gastrointestinal tract. The two major subtypes of IBD are ulcerative colitis (UC) and Crohn’s disease (CD)^1^. As the occurrence of IBD is continuously increasing, particularly in developing countries, this disease has become a concern worldwide^2^. There are two main types of treatments for IBD: induction therapy and maintenance therapy. Currently, mucosal healing is regarded as a new therapeutic goal for reducing the rate of re-hospitalizations, operation, and disability^3,4^. Commonly prescribed drugs for IBD include 5-aminosalicylic acid (5-ASA), corticosteroids, immunosuppressants, thalidomide, and biologicals^5,6^. However, these therapies cause adverse reactions, lead to a poor quality of life, and cannot achieve the treatment goals for IBD^7,8^. The etiology of IBD is not completely understood. Understanding the pathogenesis of IBD will help to explore better therapies and reduce the burden on the healthcare system. Increasing evidence has shown that IBD is likely the result of the reciprocal action between genetic factors, environmental factors, and the gut microbiome^9^.

Recently, scientists have determined the functions of many noncoding RNAs (ncRNAs) in IBD pathogenesis. Approximately 240 risk loci related to IBD have been identified in Genome Wide Association Study^10^. Most IBD-associated genetic loci are located outside of protein-coding regions and appear to affect ncRNAs^11^. ncRNAs are indispensable gene regulators at the transcriptional and translational levels and are related to IBD pathobiology^12^. ncRNAs are chiefly composed of microRNA (miRNA), long noncoding RNA (lncRNA), and circular RNA (circRNA). Among them, miRNAs have been the most thoroughly studied and numerous altered expression profiles of miRNA have been found in IBD^13^. Moreover, miRNAs are associated with inflammatory pathways in IBD, such as cytokine and chemokine regulation, deregulated autophagy, intestinal epithelial permeability, and necrosis factor-ĸB (NF-ĸB) activation^14–17^. We also determined that miRNA can be used in clinical assessment and to regulate intestinal barrier function in patients with IBD^18–20^.

Although lncRNAs and circRNAs have not been thoroughly explored in IBD, they play a crucial role in tumor angiogenesis and carcinogenesis^21–24^. Additionally, various lncRNAs and circRNAs have been identified as biomarkers for tumor diagnosis and prognosis^25–27^. Furthermore, some studies suggested that lncRNAs and circRNAs could be promising therapeutic targets of multiple diseases^28–31^. Several strategies have been proposed based on the roles of lncRNAs and circRNAs^32–35^. Of these methods, antisense oligos^36^, RNA interference^37^, small molecules^38^, and the CRISPR-Cas9 system^39^ are the leading candidates. ABX464, a small-molecule drug targeting lncRNA 0599–205, has strong anti-inflammatory effects in the dextran sulfate sodium (DSS)-induced colitis model^40^. lncRNA and circRNA will clearly become the focus of research on IBD.

In this review, we summarize the current knowledge of the molecular functions and roles of lncRNAs and circRNAs in IBD.

## Molecular functions of lncRNAs and circRNAs

lncRNAs are long-chain (more than 200 nucleotides) non-coding RNAs that lack open reading frames^41^. lncRNAs can regulate gene expression transcriptionally, post-transcriptionally, or by guiding chromatin-modifying complexes into specific genomic loci^42^. There are four major mechanisms of action encompassing most discovered mechanisms. First, lncRNAs can serve as signals to determine the time and location of gene regulation^41^. They can react with diverse stimuli and reliably reflect the actions of signaling pathways or transcription factors^41^. Moreover, lncRNAs can serve as decoys, integrating with DNA-binding proteins to avoid combining with DNA recognition elements or interacting with miRNA, thus reducing the degradation of peculiar RNA^43^. Additionally, lncRNAs can serve as scaffolds, joining several proteins to generate ribonucleoprotein complexes (lncRNA-RNPs)^43^. In many diverse biological processes, lncRNA acts as a central platform, maintaining precise control over intermolecular interactions and signaling events^43^. Finally, lncRNAs can serve as guides, directing the specific protein complexes to position at specific targets^43^. lncRNAs can control target gene expression and generate both neighboring and distant genetic changes^43^. However, an individual lncRNA may possess several mechanisms of action, and each mode is not necessarily exclusive^41^.

circRNAs are stable, evolutionarily conserved, and single-stranded RNA molecules. Unlike linear RNAs, circRNAs are closed-loop type RNAs with joined 3′ and 5′ ends^44^. There are four types of circRNAs, namely exonic circRNAs (ecircRNAs), circular intronic RNAs (ciRNAs), exon–intron circRNAs (EIciRNAs), and intergenic circRNAs^45^. circRNAs carry out parental gene transcription in addition to a small amount of protein coding^46,47^. As miRNA sponges, they can regulate RNA expression by adsorbing miRNAs^48^. Furthermore, circRNAs can interact with RNA-binding proteins to influence certain physiological processes^49^. circRNAs also act as gene transcription regulators^50^. ecircRNAs are biosynthesized by back-splicing and affect the transcription of linear RNAs which is carried out by canonical splicing^51^. Back-splicing competes with canonical splicing when these processes require the same exon and site^51^. In addition, ecircRNA likely acts as an “mRNA trap” because of isolation of the translation start site and inhibition of RNA translation^52^. ciRNAs and EIciRNAs interact with polymerase II complex and regulate the expression of parental genes, which is a prerequisite for the other functions of these RNAs^50^. ecircRNAs are found in the cytoplasm, and some can be loaded into ribosomes, which subsequently translate RNA into peptides or proteins^53^.

## Roles of lncRNAs in IBD

Although numerous studies on IBD have focused on coding-protein genes, lncRNAs are also highly expressed in IBD patients^54–56^ compared with healthy controls. Cumulative data from Genome Wide Association Study revealed a connection between IBD and lncRNA polymorphisms^57^. Although the functions of lncRNAs in pathological processes and diseases development are less well-studied compared with those in miRNAs, some of lncRNAs are related to IBD pathogenesis **(**Table 1**)**.

**Table 1:** lncRNAs significantly involved in IBD.

Classification	Disease	Source	Change	Method	Transcript/gene name	Mechanism	Ref.
LncRNA	CD	Plasma	Upgrade	Microarray	ENST00000466668	/	^[54]^
					ENST00000422548		
					ENST00000502712		
					ENST00000425364		
					NR_037605		
					ENST00000562996		
					NR_038927		
					TCONS_00014043		
					TCONS_00012771		
					ENST00000569039		
			Downgrade		uc001ody.3		
					ENST00000575787		
					uc010bmo.1		
					ENST00000509252		
					ENST00000413954		
					ENST00000431104		
					uc011dhd.2		
					TCONS_00020749		
					NR_027074		
					TCONS_00027621		
LncRNA	UC	Colonic tissues	Upgrade	Microarray	ENST00000460164.1	/	^[55]^
					ENST00000532855.1		
					ENST00000326227.5		
					ENST00000419897.1		
					ENST00000429315.2		
					ENST00000526690.1		
					ENST00000524555.1		
					ENST00000476886.1		
					ENST00000517774.1		
					ENST00000578280.1		
			Downgrade		ENST00000422420.1		
					ENST00000428597.1		
					ENST00000585267.1		
					ENST00000580576.1		
					ENST00000577551.1		
					ENST00000581051.1		
					ENST00000582072.1		
					ENST00000401008.2		
					ENST00000432658.1		
					ENST00000421632.1		
	CD		Upgrade		ENST00000460164.1		
					ENST00000532855.1		
					ENST00000326227.5		
					ENST00000419897.1		
					ENST00000520185.1		
					ENST00000526690.1		
					ENST00000445003.1		
					ENST00000522970.1		
					ENST00000524555.1		
					ENST00000429315.2		
			Downgrade		ENST00000432658.1		
					ENST00000401008.2		
					ENST00000553575.1		
					ENST00000554694.1		
					ENST00000557532.1		
					ENST00000557109.1		
					ENST00000422420.1		
					ENST00000428597.1		
					ENST00000554441.1		
					ENST00000554735.1		
LncRNA	UC	Colonic tissues	Upgrade	Microarray	BC012900	/	^[56]^
					AK001903		
					AK023330		
			Downgrade		BC029135		
					CDKN2B-AS1		
					BC062296		
LncRNA	UC	Colonic tissues	Upgrade	Microarray	BC012900	Regulated intestinal epithelial cells apoptosis	^[56]^
LncRNA	DSS-induced colitis	Mice serum and tissues	Upgrade	qPCR	NEAT1	Modulated intestinal epithelial barrier	^[64]^
LncRNA	DSS-induced colitis	Mice colonic tissues	Downgrade	Microarray	NEAT1	Regulated by 5-ALA and involved in PDT therapy treated colitis	^[68]^
LncRNA	UC	Colonic tissues	Upgrade	RNAseq	H19	Promoted mucosal regeneration	^[71]^
LncRNA	/	Mice small intestinal and colonic tissues	Upgrade	qPCR	H19	Regulated intestinal epithelial barrier	^[72]^
LncRNA	UC	Colonic tissues	Upgrade	qPCR	H19	Disrupted intestinal epithelial barrier function	^[77]^
LncRNA	/	Colonic tissues	Downgrade	qPCR	SPRY4-IT1	Regulated intestinal epithelial barrier function	^[81]^
LncRNA	DSS-induced colitis	Mice colonic tissues	Upgrade	qPCR	CRNDE	Promoted epithelial cells apoptosis	^[84]^
LncRNA	UC	Colonic tissues	Downgrade	Microarray	CDKN2B-AS1	Enhanced the barrier formation	^[88]^
LncRNA	/	Mice small intestinal tissues	Upgrade	Microarray	uc.173	Stimulated intestinal epithelium renewal	^[89]^
LncRNA	DSS-induced injury	Intestinal epithelial barrier models	Upgrade	qPCR	PlncRNA1	Regulated tight junction proteins	^[90]^
LncRNA	UC & CD	Colonic tissues	Upgrade	RNAseq	CCAT1	Increased barrier permeability	^[91]^
LncRNA	UC	Colonic tissues	Upgrade	Microarray	IFNG-AS1	Enhanced inflammation	^[99]^
LncRNA	UC	Colonic tissues	Upgrade	Microarray	IFNG-AS1	Regulated pro-inflammatory cascade	^[100]^
LncRNA	CD	Blood	Upgrade	Microarray	DQ786243	Affected CREB and Foxp3 expression and regulated Tregs function	^[103]^
LncRNA	/	Peripheral blood mononuclear cells	Downgrade	RNAseq	LINC01882	Involved in T cells activation and IL-2 expression	^[107]^
LncRNA	/	Blood and monocytes	Upgrade	RNAseq	ROCKI	Promoted inflammatory cytokines and chemokines production	^[108]^
LncRNA	/	Mice colonic tissues	Upgrade	Microarray	HIF1A-AS2	Negatively regulated intestinal inflammation	^[109]^
LncRNA	UC	Colonic tissues	Upgrade	qPCR	ANRIL	Promoted inflammatory cytokines and chemokines production	^[111]^
LncRNA	CD	Ileal tissues	Upgrade	LncRNA chip	ENST00000487539.1_1	Involved in the pathogenesis of CD	^[112]^
					ENST00000409569.2_1		
					ENST00000392442.6_1		
			Downgrade		ENST00000524613.5_1		
					ENST00000465605.5_1		

*lncRNA* long noncoding RNA, *IBD* inflammatory bowel disease, *CD* Crohn’s Disease, *UC* Ulcerative Colitis, *DSS* dextran sulfate sodium, *qPCR* quantitative real-time PCR, *NEAT1* nuclear paraspeckle assembly transcript 1, *5-ALA* 5-aminolevulinic acid, *PDT* photodynamic therapy, *RNAseq* RNA sequencing, *CRNDE* colorectal neoplasia differentially expressed, *CCAT1* colon cancer–associated transcript–1, *CREB* cAMP response element binding protein, *Foxp3* Forkhead box P3, *IL-2* Interleukin-2.

### lncRNAs and intestinal epithelial barrier dysregulation

Intestinal epithelial cells (IECs) array and make up intestinal barrier to block a variety of noxious substances such as the microbiota, microbial products, and antigens in the lumen. The specialized structures in the intestinal barrier comprise tight junctions (TJs) and adherent junctions (AJs), ensuring the function of the epithelial barrier^58^. Studies in patients with IBD showed that intestinal barrier function is disrupted in both active and quiescent disease states^59,60^. Furthermore, disrupted intestinal barrier, reduction of junctional proteins, and increased intestinal permeability were observed in patients with CD^61^. Increased epithelial permeability has also been observed in the inactive phase and is strongly predictive of clinical relapse. Destruction of the epithelial barrier is an initial characteristic of disease relapse, suggesting that it plays an initiating role of mucosal inflammation. Many studies have revealed the connection between lncRNAs and the intestinal epithelial barrier.

#### NEAT1

lncRNA nuclear paraspeckle assembly transcript 1 (NEAT1) is an inflammatory cytokine regulator related to the innate immune response^62^. NEAT1 is also a key component of the ribonucleoprotein complexes regulating DNA-mediated activation of the innate immune response^63^. Liu et al.^64^ reported that compared with control groups, NEAT1 was over-expressed in the intestinal tissues, serum, and exosomes of DSS-induced mice, and in tumor necrosis factor (TNF)-α-induced inflammatory cell models. Similarly, epithelial cell permeability was increased in the above mice and cell models compared with in control groups^64^. NEAT1 suppression reversed the effects in TNF-α- and DSS-induced IBD models, decreased epithelial cells permeability, and enhanced intestinal epithelial integrity^64^. However, Birkl et al.^65^ found that TNF-α may be essential for mucosa repair in the early stage of inflammation. NEAT1 suppression also promoted macrophage polarization towards alternatively activated macrophages (“M2”) rather than classically activated macrophages (“M1”) and inhibited inflammation^64^. These results revealed that NEAT1 is involved in IBD pathogenesis by regulating intestinal epithelial barrier function and the key mediators involved in disease should be precisely targeted during treatment. Photodynamic therapy (PDT) is a promising therapy for IBD^66^, particularly low-dose PDT. Farve et al.^67^ demonstrated that delta-aminolevulinic acid (δ-ALA)-induced low-dose PDT alleviated T-cell-mediated mice colitis and adverse events were negligible. Wang et al.^68^ verified that 5-ALA-induced PDT relieved DSS-induced colitis in mice through the NEAT1-miRNA204–5p axis.

#### H19

lncRNA H19 is transcribed from the H19 gene on chromosome 11 (Brannan et al.^69^). H19 can be found in multiple tissues during the embryonic stage but is silenced after birth^70^. Under extensive pathological conditions, H19 over-expression is universally detected. Intestinal H19 was dramatically upregulated in mice colitis models, as well as in inflamed colonic tissues from patients with IBD^71^. Inflammation-induced H19 was observed in IECs. H19 induced by the inflammatory cytokine IL-22 promoted IEC proliferation, epithelial regeneration, and mucosal healing^71^. Mechanistically, H19 antagonized negative regulators of IECs proliferation, such as p53 protein, miRNA-34a, and let-7, and increased the expression of multiple cell growth-promoting genes in the epithelium^71^. Other researchers also observed the negative effects of H19. Highly expressed H19 repressed the function of mRNAs encoding TJ protein ZO-1 and AJ protein E-cadherin by releasing miR-675, leading to epithelial barrier damage^72^. HuR over-expression prevented miR-675 from releasing from H19, promoted ZO-1 and E-cadherin generation, and eliminated H19-induced barrier malfunction^72^. In contrast, targeted HuR deletion increased the abundance of miR-675 in the intestinal barrier and postponed the recovery of intestinal barrier in mice suffering from pathological stimulus^72^.

Vitamin D receptor (VDR) is a receptor of 1,25 (OH)_2_D3 in humans^73^. 1,25(OH)_2_D3 is the Vitamin D active form^73^ and prevents gut damaged by certain destructive reagents^74^. In multiple tissues, VDR plays an important role in regulating inflammation and carcinogenesis^75,76^. lncRNA H19 over-expression in UC tissues may decrease VDR and disrupt intestinal epithelial barrier function which is involved in the development of UC^77^. H19 over-expression significantly decreased ZO-1, occludin, and VDR levels and impaired the function of the Caco-2 monolayer barrier^77^. The disruptive effect of H19 was partly due to miR-675–5p, which targeted the 3′-untranslated region of VDR mRNA^77^. miR-675–5p inhibitors can increase ZO-1 and VDR levels^77^. Therefore, the interaction between lncRNA H19 and VDR signaling may contribute to studies on therapeutic targets for UC.

#### SPRY4-IT1

Transcribed from the SPRY4 gene, lncRNA SPRY4-IT1 does not code for proteins^78^. The RNA-binding proteins HuR acts as critical regulator of TJ proteins in the intestine, and HuR dysregulation results in disruption of the epithelial barrier in vitro and in vivo^79,80^. The 3′-untranslated regions of mRNAs encoding the TJ proteins such as claudin-1, -3, occludin, and JAM-1 include several SPRY4-IT1-binding sites, SPRY4-IT1 silencing caused TJ mRNAs to shift from high-translating sections to low-translating sections in polyribosomes^81^. SPRY4-IT1 pull-down led to intestinal epithelial barrier dysfunction by reducing the stability of TJ mRNAs^81^. The process of SPRY4-IT1 regulated TJ mRNAs was enhanced when SPRY4-IT1 associated with HuR^81^. However, SPRY4-IT1 can interact with TJ mRNAs directly without interacting with HuR^81^. HuR silencing decreased rather than obstructed the association of SPRY4-IT1 with these TJ mRNAs^81^. Increasing SPRY4-IT1 levels in the gut showed protective effects by increasing TJ protein expression^81^. As patients with IBD have increased intestinal permeability, novel molecular therapies aiming to over-express lncRNA SPRY4-IT1 may control gut permeability in specific clinical settings.

#### CRNDE

The lncRNA colorectal neoplasia differentially expressed (CRNDE) was highly expressed in colorectal adenomas and carcinomas^82^. CRNDE may be involved in tumorigenesis by regulating miRNAs^83^. Yang et al.^84^ suggested that CRNDE is also associated with IBD progression. CRNDE was highly expressed in tissues from DSS-induced mice colitis and human colonic epithelial cells models^84^. CRNDE inhibition reduced DSS-induced cell apoptosis and cleaved caspase-3, and the apoptotic rate was significantly decreased^84^. In DSS-induced cell models, CRNDE suppressed miRNA-495 and increased suppressor of cytokine signaling (SOCS1)^84^. miRNA-495 has been found to be decreased in UC and prevented IEC apoptosis through the JAK signaling pathway^85^. SOCS1 restricted cytokine receptor signaling^86^ and promoted IFN-γ-induced IEC apoptosis^87^. The CRNDE/miR-495/SOCS1 axis was also validated in DSS-induced mice colitis models^84^. The clinical features of these mice were alleviated after interfering with CRNDE expression, showing improvement in body weight loss and a reduction in bloody stools^84^. Therefore, lncRNA CRNDE is a potential target for regulating IECs apoptosis through the CRNDE/miR-495/SOCS1 axis.

#### Other lncRNAs

The lncRNA CDKN2B-AS1 has more than 20 spliced variants containing canonical spliced linear RNA and back-spliced circular RNA molecules^88^. The longest linear and major circular RNA shape of CDKN2B-AS1 were decreased in UC colon tissues^88^. Reducing the levels of both linear and circular CDKN2B-AS1 enhanced the barrier formation ability of colonic epithelium by disrupting Claudin-2 expression^88^. A reduction in CDKN2B-AS1 improved barrier function, showing that the absence of CDKN2B-AS1 in patients may play a protective role after damage^88^. Xiao et al.^89^ discovered that elevation of lncRNA uc.173 promoted intestinal epithelium growth. Reduced uc.173 levels slowed IEC renewal by interacting with the pri-miR-195 transcript, resulting in miRNA-195 degradation^89^. lncRNA BC012900 over-expression resulted in inhibition of IEC proliferation and increased the susceptibility of these cells to apoptosis^56^. This likely occurred by increasing the abundance of PPM1A (protein phosphatase, Mg^2+^/Mn^2+^-dependent, 1A)^56^. Chen et al.^90^ reported that elevated PlncRNA1 levels prevent intestinal epithelial barrier injury. Furthermore, PlncRNA1 regulates the level of miRNA-34c^90^. These two ncRNAs supported the regular effect of the intestinal barrier by mediating the production of TJ proteins ZO-1 and occludin, as well as MAZ^90^. Another lncRNA, colon cancer–associated transcript–1 (CCAT1), over-expressed in IBD tissues compared with in normal tissues and may be associated with the development of IBD^91^. CCAT1 can serve as miRNA-185–3p sponge and maintain the stability of myosin light chain kinase (MLCK) mRNA by decreasing miRNA-185–3p binding to MLCK mRNA in Caco-2 cells^91^. MLCK and its phosphorylation product regulated TJs assembly and increased intestinal permeability^91^. The positive correlation between CCAT1 and MLCK accelerates IBD development^91^.

### lncRNAs and immune homeostasis dysregulation

IBD is an inflammation disease of the intestinal mucosa, as well as a sustained and aberrant immune disorder, caused by defects in the regulation of intestinal mucosal immunity^92^. NF-ĸB is a representative immune response factor that can translocate into the nucleus when NF-ĸB inhibitory protein is phosphorylated and then degraded^93^. This results the transcription of target genes such as interleukin-1β (IL-1β), interleukin-8 (IL-8), and interferon-γ (IFN-γ)^94^. Some studies reported that excessive inflammatory incidents, such as NF-ĸB activation and high pro-inflammatory cytokines expression, contribute to colitis^95,96^. Excessive accumulation of immune cells in the gut and induction of complex inflammatory networks make it difficult to explain the roles of individual cytokines and immune pathways as well as the precise etiology and the pathogenesis of IBD^97^. Interleukin-1b, IL-6, IL-8, and TNF stimulate NF-ĸB, which triggers the transcription of pro-inflammatory cytokines. Regulatory T lymphocytes (Tregs) are an important subset of T lymphocytes. They can limit the functions of immune cells and maintain immunity. Treg dysfunction is attributed to CD and disease severity^98^.

#### IFNG-AS1

The lncRNA IFNG-AS1 is located at chromosome 12 in human and is close to *IFNG*. IFNG-AS1 was increased in patients with active UC compared with in both healthy controls and non-inflamed tissues of patients with UC^56^. Using human UC samples, mice colitis models, and Jurkat T cell models, Padua et al. found that IFNG-AS1 was related to the IBD single nucleotide polymorphism (SNP) rs7134599 (Padua et al.^99^). There is a positive link between IFNG-AS1 over-expression and the crucial inflammatory cytokine IFNG expression in immune cells^99^. Moreover, Rankin et al.^100^ illustrated that the IFNG-AS1 gene is located beside the inflammatory cytokine IL-22 gene and extensively regulates the pro-inflammatory cascade. IFNG-AS1 may promote the effects of Th1 cytokines (IFNG, IL-2) and reduce the effects of Th2 cytokines (IL-10, IL-13) through an MLL/SET1 mechanism^100^. Overall, lncRNA IFNG-AS1 is a potential target for treating patients with colitis.

#### DQ786243

Tregs dysfunction is involved in CD and its severity^98^. Forkhead box P3 (Foxp3) and cAMP response element-binding protein (CREB) are transcription factors required for the generation, function, and development of Tregs^101,102^. Zhang et al.^103^ discovered that the expression of the lncRNA DQ786243 and CREB were increased in the blood of patients with active CD compared with those in the inactive CD and healthy controls. Interestingly, Foxp3 expression was decreased in the blood of patients with inactive CD compared with that in active CD or healthy controls^103^. DQ786243 may have a significant effect on regulating CREB and Foxp3 genes^103^. DQ786243 transfection in Jurkat cells promoted CREB and Foxp3 expression as well as CREB phosphorylation in vitro^103^. As the expression of CREB and Foxp3 in the blood of patients with CD is not significantly correlated, CREB phosphorylation rather than CREB itself may affect Foxp3 expression^103^. Moreover, the DQ786243, CREB, and Foxp3 mRNAs are related to C-reactive protein (CRP), which is a vital serum biomarker of inflammation^103^. These finding suggest that lncRNA DQ786243 is involved in CD pathogenesis and may regulate Tregs function by affecting CREB and Foxp3 expression.

#### LINC01882

There is variation in the genetic locus of protein tyrosine phosphatase 2 (PTPN2) in IBD^104^. PTPN2 regulates cytokines signaling by acting on multiple phosphorylated proteins^105^. A study of patients with CD demonstrated a link between the SNP rs2542151 and lower levels of PTPN2 protein in colonic fibroblasts, as well as the formation of aberrant autophagosomes in IECs^106^. PTPN2 locus SNPs are related to changes in the lncRNA LINC01882, which is primary expressed in T cells and involved in autoimmune diseases, including IBD^107^. LINC01882 may participate in IL-2 expression, which affects differentiation, immune responses, and homeostasis of various lymphocytes, including Tregs^107^. Changes in the number of Tregs can contribute to the progress of autoimmune diseases^107^. However, this study mainly focused on rheumatoid arthritis, and the relationship between LINC01882 and IBD requires further analysis.

#### Other lncRNAs

The lncRNA ROCKI negatively regulated its cognate encoding gene, myristoylated alanine-rich protein kinase C (MARCKS), by constituting a compound at the MARCKS promoter, which then promoted inflammatory cytokine and chemokine production^108^. The expression of MARCKS, mediated by ROCKI, may contribute to IBD^108^. Quan et al.^109^ examined *Roseburia intestinalis* flagellin-induced lncRNA expression profiles and found that lncRNA HIF1A-AS2 inactivated the NF-ĸB/JNK pathway and decreased the expression of cytokines IL-1β, IL-6, IL-12, and TNF-α. HIF1A-AS2 was effective for alleviating inflammatory responses in vitro and in vivo; therefore, HIF1A-AS2 may be a negative modulator of intestinal inflammation. The lncRNA ANRIL located at chromosome 9p21 is significantly downregulated in patients with UC^110^. ANRIL inhibition remarkably reversed the effects of injury by improving cell viability, suppressing cell apoptosis, and reducing inflammatory cytokine production^111^. The suppressive effects of lncRNA ANRIL were achieved through the TLR4/MyD88/NF-ĸB pathway, which further inhibited UC development^111^. Li et al.^112^ identified numerous lncRNAs differentially expressed in the mucosa of CD and predicted a lncRNA-miRNA/TF mRNA network. Most of these lncRNAs are related to cell signaling pathways and immune reactions^112^. This network is conductive to improve the efficiency of CD gene searches and provides a foundation for follow-up studies^112^.

### lncRNAs as IBD biomarkers

IBD has a large influence on the quality of life and health care system. Clinical manifestations, endoscopic evaluation, imaging methods, and histopathological examinations are commonly used in IBD treatment. However, the clinical features of IBD differ between individuals, and ~25% patients have extraintestinal features before diagnosis^113^. Endoscopy and histopathological examinations are known as the “gold standard” of IBD diagnosis^114,115^. But both approaches heavily rely on skilled clinicians, and many adopt alternative methods as a result^116^. These factors all contribute to the difficulty of diagnosis. Consequently, researchers prefer the use of biomarkers, such as C-reactive protein (CRP), calprotectin, lactoferrin, and others. However, sensitive and specific biomarkers for IBD are lacking. Many lncRNAs have been shown to be involved in IBD. Changes in lncRNA levels can be applied for monitoring of IBD. lncRNAs should be exploited for IBD diagnosis and prognosis, as well as for predicting therapeutic responses. Many lncRNAs can serve as biomarkers for the clinical evaluation of patients with IBD (Table 2).

**Table 2:** LncRNAs/circRNAs proposed for IBD biomarkers and therapeutic predictors.

Classification	Disease	Source	Change	Method	Transcript/gene name	Application	Ref.
LncRNA	UC & CD	Colonic tissues & blood samples	Upgrade	qPCR	KIF9-AS1	Biomarker between IBD and HC	[117]
					LINC01272		
			Downgrade		DIO3OS		
LncRNA	CD	Colonic tissues	Downgrade	qPCR	ANRIL	Biomarker between CD and HC, assessed infliximab response	[118]
LncRNA	CD	Ileal tissues	Upgrade	Immunochip	RP11–679B19.1	Associated with recurrent fibrostenotic CD	[120]
LncRNA	CD	Ileal tissues	Upgrade	RNAseq	HNF4A-AS1	Associated with severe mucosal ulcers	[121]
			Downgrade		LINC01272		
LncRNA	UC & CD	Peripheral blood	Upgrade	qPCR	GAS5	Marker of glucocorticoid therapy in children	[125]
CircRNA	UC & CD	Peripheral blood mononuclear cells	Upgrade	Microarray	Circ-103516	Biomarker between IBD and HC	[134]
CircRNA	CD	Peripheral blood mononuclear cells	Upgrade	Microarray	Circ-004662	Biomarker between CD and UC, HC	[143]
					Circ-092520	Biomarker between CD and HC	
					Circ-102610		
					Circ-103124		

*lncRNA* long noncoding RNA, *circRNA* circular RNA, *IBD* inflammatory bowel disease, *UC* ulcerative colitis, *CD* Crohn’s Disease, *HC* healthy control, *qPCR* quantitative real-time PCR, *RNAseq* RNA sequencing, GAS5 growth arrest-specific 5.

### lncRNA as prognostic and diagnostic biomarkers in IBD

Wang et al.^117^ demonstrated that in tissues and plasma samples from patients with IBD, lncRNA DIO3OS was significantly downregulated whereas lncRNA KIF9-AS1 and LINC01272 were significantly upregulated compared with in healthy controls^117^. KIF9-AS1, LINC01272, and DIO3OS have latent diagnostic value for IBD^117^. The areas under the ROC curve (AUCs) between these three lncRNAs in patients with IBD and healthy controls are mostly higher than 0.76 (Wang et al.^117^). In summary, the expression of lncRNA KIF9-AS1, LINC01272, and DIO3OS in tissues and plasma samples from IBD patients differed from that in healthy controls and has potential diagnostic value for IBD detection^117^. Ge et al.^118^ illustrated that the level of lncRNA ANRIL distinguished patients with CD from healthy controls. The AUC value of ANRIL was 0.803 (Ge et al.^118^). Interestingly, lncRNA ANRIL can also distinguish the active stage of CD from the remission stage, with an AUC value of 0.839 (Ge et al.^118^). ANRIL showed negative correlations with disease risk, disease activity, and pro-inflammatory cytokines levels but positive correlations with anti-inflammatory cytokines levels^118^. lncRNAs acted as biomarkers in both early and late disease stages, even when complications were present. Over half of patients with CD develop complications over time, such as fistulae and stenosis^119^. The lncRNA RP11–679B19.1 was shown to be associated with recurrent fibrostenotic CD, but its detailed mechanism remains unknown^120^.

### lncRNAs as predictors of therapeutic response in IBD

The lncRNA ANRIL can serve as a biomarker under multiple conditions. Changes in ANRIL expression are associated with the infliximab treatment response in patients with CD. ANRIL from responders of infliximab treatment was increased, whereas that from unresponsive individuals remained stable^118^. ANRIL upregulation in the intestinal mucosa could act as a marker for assessing the response to infliximab treatment in patients with CD^118^. Haberman et al.^121^ reported that based on the intestinal biopsies of the pediatric patients with IBD under treatment, who underwent diagnostic endoscopies, lncRNA HNF4A-AS1 and LINC01272 expression was significantly correlated with severe mucosal ulcers. In addition, LINC01272 showed a significantly positive correlation with calprotectin S100A8, which is currently used as a clinical biomarker of tissue inflammation. However, HNF4A-AS1 was negatively correlated with calprotectin S100A8 (Haberman et al.^121^). LINC01272 was specifically expressed in myeloid dendritic cells (DC), monocytes, and neutrophils, whereas HNF4A-AS1 was specifically expressed in epithelial cells^121^. Tissue-specific lncRNA HNF4A-AS1 and LINC01272 expression resulted in the development of a novel lncRNA-directed therapy with fewer off-target effects^121^. Corticosteroids are commonly prescribed drugs for IBD. Glucocorticoids (GCs) with anti-inflammatory and immunosuppressive effects are used to induce remission in IBD patients^122^. However, ~20% of patients applying GCs developed resistance to GCs, and 40% of patients maintained clinical remission, relying on GCs. In poor responders to GCs, the levels of lncRNA growth arrest-specific 5 (GAS5) were higher than those in good responders; therefore, GAS5 may be associated with GCs resistance^123,124^. A later study demonstrated that the expression of GAS5 differed between GCs-sensitive and GCs-resistant cells, and GAS5 is positively correlated with GCs resistance in children with IBD^125^. Additionally, endogenous GAS5 affects GCs effectiveness, likely because it accumulates in the cytoplasm and plays a role at the post-transcriptional level^125^. Overall, lncRNA GAS5 can be considered as a novel candidate marker and shows potential for use in the personalization of GCs therapy^125^.

## Roles of circRNAs in IBD

circRNAs are related to a large number of biological processes and diseases. For example, circQTL SNPs are significantly enriched for the Genome Wide Association Study variants associated with various diseases, particularly IBD, schizophrenia, and type II diabetes mellitus^126^. As a regulator of gene expression, circRNA acts on genetic variation and phenotypic changes. Some circRNAs have been demonstrated to participate in the nosogenesis of IBD and colitis-associated cancer (CAC; Table 3). Although many circRNAs have been identified in humans, functional studies of circRNAs in IBD have not been widely conducted. Thus, analyzing alterations in circRNA profiles and their roles are likely to reveal fundamental molecular mechanisms in IBD.

**Table 3:** circRNAs significantly involved in IBD.

Classification	Disease	Source	Change	Method	Transcript/gene name	Mechanism	Ref.
CircRNA	DSS-induced colitis	Mice and human ISCs	Upgrade	Microarray	CircPan3	Improved self-renewal ability of ISCs	[128]
CircRNA	/	Mice small intestinal tissues	Upgrade	qPCR	CircPABPN1	Regulated autophagy gene expression in intestinal epithelium	[133]
CircRNA	UC & CD	Peripheral blood mononuclear cells	Upgrade	Microarray	CircRNA_103516	Mediated inflammation and immune-related signaling pathway	[134]
CircRNA	CD	Colonic tissues	Upgrade	Microarray	hsa-circRNA-102685	Involved in signaling pathways of CD	[136]
CircRNA	AOM/DSS-induced colon carcinoma	Mice colonic tissues	Upgrade	RNAseq	mmu_circRNA_001801	Involved in colitis-associated cancer	[148]
					mmu_circRNA_002987		
					mmu_circRNA_001155		
			Downgrade		mmu_circRNA_00287		
					mmu_circRNA_003037		
					mmu_circRNA_001226		

*circRNA* circular RNA, *IBD* inflammatory bowel disease, *DSS* dextran sulfate sodium, *ISCs* intestinal stem cells, *qPCR* quantitative real-time PCR, *UC* ulcerative colitis, *CD* Crohn’s disease, *AOM* azoxymethane, *RNAseq* RNA sequencing.

### circRNAs and intestinal epithelial barrier dysregulation

The intestinal epithelium is a cell monolayer constituting an important gut barrier. Intestinal stem cells (ISCs) are rapidly self-renewed and can differentiate into the intestinal epithelium^127^. Lgr5+ ISCs are an ISC subgroup. circRNA circPan3 (transcribed from *Pan3*) was over-expressed in human Lgr5+ ISCs and mouse Lgr5-GFP + ISCs^128^. circPan3 increased the level of IL-13 receptor subunit (IL-13Rα1) by binding to the mRNA of IL-13Rα1 in ISCs and improved the self-renewal capacity of ISCs^128^. circPan3 deletion in human Lgr5+ ISCs impaired ISC self-renewal and epithelium regeneration capacity^128^. Similarly, circPan3 bound to IL-13Rα1 mRNA in mice ISCs to preserve its stability and allowed IL-13Rα1 expression in these cells^128^. In summary, the self-renewal of both human and mice ISCs required the presence of circRNA circPan3 through the IL-13Rα1-mediated signaling pathway.

Although the intestinal epithelial barrier can block most pathogens, several pathogenic bacteria can escape from the barrier and invade IECs^129^. Autophagy can target and degrade cytoplasmic pathogens in lysosomes^130^. ATG16L1 is produced from the Atg16l1 gene and has a vital role in autophagy and intestinal epithelium homeostasis. HuR is an RNA-binding protein that is a vital post-transcriptional regulator in the intestinal epithelium^131,132^. HuR and circRNA circPABPN1 regulated the expression of ATG16L1 in the intestinal epithelium^133^. Highly expressed circPABPN1 repressed HuR binding to Atg16l1 mRNA in IECs, and then prevented HuR-induced ATG16L1 translation, as well as reduced ATG16L1 production^133^. The HuR interaction with circPABPN1 partly regulated autophagy by modulating ATG16L1 translation, suggesting that the HuR/circPABPN1/ATG16L1 axis is related to the nosogenesis of IBD and other mucosal disorders^133^.

### circRNAs and immune homeostasis dysregulation

circRNA_103516 was remarkably increased in the active period compared with in emission period of both CD and UC and was positively correlated with disease activity (CD activity index, Mayo, CRP, and erythrocyte sedimentation rate)^134^. In patients with circRNA_103516 was positively correlated with pro-inflammatory cytokines and negatively correlated with anti-inflammatory cytokine^134^. This suggests that circRNA_103516 carried out a pro-inflammatory function through inflammation and immune events involved in IBD. Furthermore, the AUC values of circRNA_103516 for UC and CD were 0.687 and 0.790, respectively^134^. circRNA_103516 presented substantial clinical value for CD and UC, and may therefore also be considered as a novel biomarker for IBD^134^. In patients with stricture and penetrating CD, the positive prevalence of circRNA_103516 was higher. Thus, circRNA_103516 may contribute to stricture and penetrating behavior of CD^134^. Moreover, miRNA-19b may inhibit SOCS3 to participate in IEC chemokine production^135^. circRNA_103516 was negatively correlated with hsa-miRNA-19b-1–5p in patients with CD but not with UC^134^. circRNA_103516 may be implicated in the molecular mechanisms of CD through hsa-miRNA-19b-1–5p sponging^134^.

Qiao et al.^136^ found that circRNA-102685 is highly expressed in the colon tissues of patients with CD and potentially regulates miRNA-146. miRNA-146b relieved gut inflammation by activating NF-ĸB in animal experiments^137^ and affected the functions of multiple immune cells, such as, Tregs cells and dendritic cells^138^. In addition, circRNA-102685 was involved in other pathways, such as the chemokine signaling pathway and apoptosis. Those pathways have been identified as being involved in IBD^139–141^. However, the results of Qiao et al.^136^ are not comprehensive because limited tissue specimens were examined. Generally, circRNA-102685 expression may be related to CD pathogenesis^136^.

### circRNAs as IBD biomarkers

Identifying diagnostic and prognostic biomarkers of IBD will help predict disease behavior and monitor treatment responses. circRNAs with ring structures are more stable than linear RNAs in tissues and body fluids^142^. This property suggests that circRNAs can act as promising biomarkers of IBD. Some circRNAs were found to be latent biomarkers of IBD (Table 2). Yin et al.^143^ demonstrated that four circRNAs (004662, 092520, 102610, and 103124) were significantly upregulated in peripheral blood mononuclear cells of patients with CD compared with that those in healthy controls. Furthermore, compared with patients with UC, circRNA_004662 showed higher expression in patients with CD^143^. The AUC values of these four circRNAs (092520, 102610, 004662, and 103124) were 0.66, 0.78, 0.85, and 0.74, respectively, making these circRNAs potential diagnostic biomarkers of CD^143^. Considering the observed diagnosis values (*P*-value, sensitivity, specificity, and AUC), circRNA_004662 may emerge as a promising biomarker to differentiate CD from UC^143^. circRNA_004662 was an ecircRNA back-spliced from the exon of superoxide dismutase 2 gene and protected cells by mitochondrial reactive oxygen species detoxification^144^. circRNA_004662 may be related to the mammalian target of rapamycin pathway which limits pro-inflammatory cytokines and enhances anti-inflammatory responses^145^.

### circRNAs in CAC

The CAC risk is increased in patients with IBD, which is in directly linked to the extent and duration of inflammation^146^. circRNAs with diverse functions are new hotspots for researchers studying the noncoding cancer genome^147^. The altered expression profiles of circRNAs may be correlated with CAC at the transcription level.

Yuan et al.^148^ reported that altered expression profiles of circRNAs are involved in CAC development. Compared with in normal colonic tissues, mmu_circRNA_001801, mmu_circRNA_002987, and mmu_circRNA_001155 were the most increased circRNAs, and mmu_circRNA_00287, mmu_circRNA_003037, and mmu_circRNA_001226 were the most decreased circRNAs^148^. Moreover, this study explored the possible connections between circRNAs and miRNAs and attempted to establish a network. It was found that mmu_ circRNA_001226 and mmu_circRNA_000287 were the first two key points in this network^148^. The mmu-circ-001226/mmu-circ-000287-miRNA-mRNA network may be the potential mechanism for CAC^148^.

## Conclusion

**Fig. 1:**
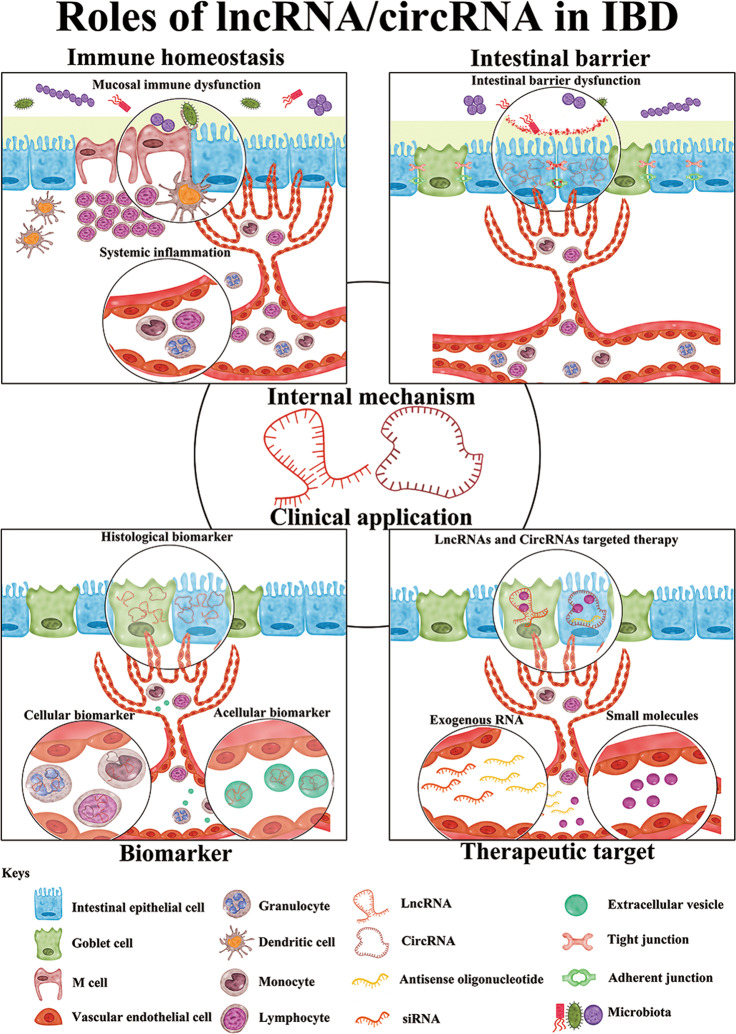
Roles of lncRNA/circRNA in IBD. Roles of lncRNA/circRNA in IBD are roughly classified into internal mechanisms and clinical applications. Internal mechanisms mainly focus on lncRNAs/circRNAs regulating immune homeostasis and the intestinal barrier. Clinical applications mainly include biomarkers and therapeutic predictors. lncRNAs/circRNAs can also function as therapeutic targets of IBD with continued research and technique development. lncRNA long noncoding RNA, circRNA circular RNA, IBD inflammatory bowel disease, siRNA small interfering RNA.

For the past few decades, IBD has become a global health concern^149^. Its precise pathogenesis remains incompletely understood. Although research on lncRNAs and circRNAs in IBD is still in the early stages, many lncRNAs and circRNAs have been implicated in IBD pathogenesis and have shown promising prospects for clinical applications (Fig. 1). Multiple aspects of lncRNAs, circRNAs, and their functions in IBD require further investigation. Technical tools used to identify the biological mechanism of ncRNAs in human IBD should be improved. Moreover, the complexity of IBD nosogenesis and limited available information indicated that a single lncRNA or circRNA may not entirely explain IBD. Based on the close interactions between lncRNAs, circRNAs, and IBD, it is crucial to further elucidate the molecular mechanisms of these RNAs in IBD, in addition exploring promising therapeutic approaches.

Inhibition or enhancement of lncRNAs and circRNAs may be useful for IBD treatment. For efficiency, the development of antagonists or mimics of lncRNAs and circRNAs must be based on tissue-specific or cell type-specific characteristic. Rigorous clinical trials are required to assess the effect and security of these promising treatments. In summary, lncRNAs and circRNAs are promising areas of research for investigating IBD pathogenesis and potential clinical applications.
